# Neuroimaging Studies on Disorders of Consciousness: A Meta-Analytic Evaluation

**DOI:** 10.3390/jcm8040516

**Published:** 2019-04-16

**Authors:** Manuela Berlingeri, Francesca Giulia Magnani, Gerardo Salvato, Mario Rosanova, Gabriella Bottini

**Affiliations:** 1 Department of Humanistic Studies (DISTUM), University of Urbino Carlo Bo, 61029 Urbino, Italy; 2 Center of Clinical Developmental Neuropsychology, ASUR Marche, Area Vasta 1 Pesaro, 61122 Pesaro, Italy; 3 NeuroMi, Milan Center for Neuroscience, 20126 Milano, Italy; francescag.magnani@gmail.com (F.G.M.); gerardosalvato@gmail.com (G.S.); g.bottini@unipv.it (G.B.); 4 Center of Cognitive Neuropsychology, ASTT Grande Ospedale Metropolitano Niguarda, 20162 Milano, Italy; 5 Brain and Behavioral Science Department, Università degli Studi di Pavia, 27100 Pavia, Italy; 6 Department of Biomedical and Clinical Sciences “Luigi Sacco”, University of Milan, 20122 Milano, Italy; mario.rosanova@unimi.it; 7 Fondazione Europea di Ricerca Biomedica Onlus, 20063 Milan, Italy

**Keywords:** fMRI, minimally conscious state, unresponsive wakefulness syndrome, GingerALE

## Abstract

Neuroimaging tools could open a window on residual neurofunctional activity in the absence of detectable behavioural responses in patients with disorders of consciousness (DOC). Nevertheless, the literature on this topic is characterised by a large heterogeneity of paradigms and methodological approaches that can undermine the reproducibility of the results. To explicitly test whether task-related functional magnetic resonance imaging (fMRI) can be used to systematically detect neurofunctional differences between different classes of DOC, and whether these differences are related with a specific category of cognitive tasks (either active or passive), we meta-analyzed 22 neuroimaging studies published between 2005 and 2017 using the Activation Likelihood Estimate method. The results showed that: (1) active and passive tasks rely on well-segregated patterns of activations; (2) both unresponsive wakeful syndrome and patients in minimally conscious state activated a large portion of the dorsal-attentional network; (3) shared activations between patients fell mainly in the passive activation map (7492 voxels), while only 48 voxels fell in a subcortical region of the active-map. Our results suggest that DOCs can be described along a continuum—rather than as separated clinical categories—and characterised by a widespread dysfunction of brain networks rather than by the impairment of a well functionally anatomically defined one.

## 1. Introduction

Severe brain injuries can lead to Disorders of Consciousness (DOC), a variety of neurological conditions ranging from the unresponsive wakefulness syndrome (UWS), previously known as vegetative state, to the Minimally Conscious State (MCS) [1,2,3]. Diagnosis of DOC relies on the use of standard behavioural scales, such as the Coma Recovery Scale-revised (CRS-R) [4,5]. Specifically, the use of these standardised tools significantly ameliorated the reliability of the diagnosis in the clinical routine: up to the 41% of patients diagnosed as UWS, for example, could be actually classified as MCS when assessed by means of the CRS-R [6]. This demonstrates that increasing the diagnostic power for DOC may relevantly improve prognosis and selection of treatments, and facilitate end-of-life decisions. Nevertheless, the reliability of behavioural scales strongly depends on the integrity of patients’ brain systems for sensorimotor, attention, memory, and language functions, so that neuroimaging and electrophysiological tools are needed to allow and ameliorate the diagnosis in DOC patients. Indeed, these instruments may provide objective assessments of the residual brain activity, both at rest and during specific tasks, also in the absence of behavioural responses. Specifically, active and passive paradigms have been adopted to detect the patients’ residual ability to generate meaningful neural activations in response to environmental stimuli in the context of functional magnetic resonance imaging (fMRI) studies. A classic example of the distinction between active and passive tasks can be found in the study by Owen et al. [7], in which neural responses to a passive sentence listening task were compared with neural responses to active mental imagery task. However, the neuroimaging studies published so far have only provided anecdotal results as, for the majority of the experiments, only small groups of patients have been investigated, without providing a comparison with healthy controls. Therefore, to support this interesting evidence, a systematic replication of the fMRI results that goes beyond the specific experimental settings and paradigms adopted by every single study is needed. 

We aimed at formally addressing this issue by meta-analysing the fMRI/PET results available in the international literature with the Activation Likelihood Estimate (ALE) method initially developed by Turkeltaub et al. [8], which represents one of the most adopted approaches for the quantitative meta-analysis of neuroimaging data. We searched for the brain areas associated with active and passive tasks in DOC patients classified as UWS and with MCS. We hypothesised that:

1. active and passive tasks would share brain regions associated with low-level cognitive processing, e.g., auditory processing, while they should be anatomically segregated at the level of higher-order-associative cortices;
2. UWS and MCS patients would be characterised by the activation of dissociable neural networks. In particular, we expect that the dorsal-attentional network, responsible for externally directed cognitive processes, would be mainly recruited in MCSs;
3. UWS and MCS activations would overlap in regions associated with low-level cognitive tasks, i.e., with passive tasks mainly, while no signs of shared activations should be found in brain regions recruited during active tasks.

## 2. Experimental Section

### 2.1. Study Selection

**Figure 1:**
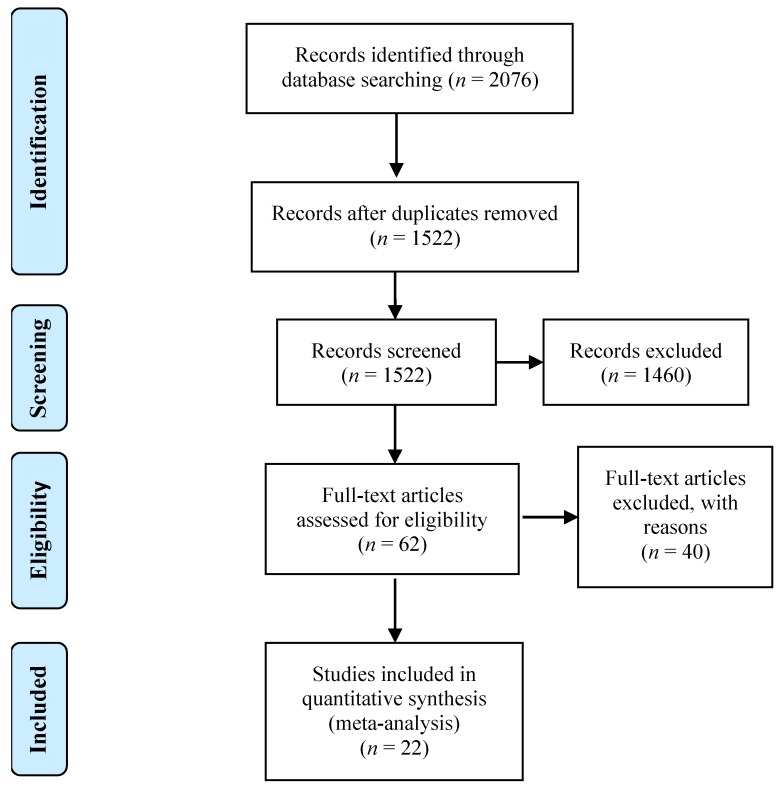
Flow diagram of meta-analysis. The figure shows the entire flow of studies selection (from [12]).

Our meta-analysis is based on 22 fMRI studies published between 2005 and 2017 which investigated the residual neurofunctional activations in severely brain-damaged patients with DOC (either UWS (previously known as “vegetative state”) or MCS). Studies were selected by querying the following key words in PubMed (http://www.ncbi.nlm.nih.gov/pubmed/): “*vegetative state* AND fMRI”, “*minimally conscious state* AND fMRI”, “*disorders of consciousness* AND fMRI”; “*vegetative state* AND PET”, “*minimally conscious state* AND PET”, “*disorders of consciousness* AND PET”. Among all the retrieved records, we excluded duplicates and non-pertinent studies. We did not include, for example, studies using other techniques such as VBM or DTI, or studies on functional connectivity. The 22 imaging selected studies met the following criteria: (1) activation fMRI-PET paradigms; (2) no resting state fMRI; (3) only data emerging from classical massively univariate analyses; (4) reporting either the stereotactic coordinates, or the anatomical label of the activated region either in the radiological, or in the neurological convention according to the AAL [9]. It is worth noting that in some cases this represented an issue; for example we had to exclude the study by Schiff et al. [10] and Zhu et al. [11] because they reported the coordinates in the Talairach system, but in a radiological convention. As a consequence, by flipping the coordinates along the y-axis (to obtain coordinates in neurological convention) and then transforming the TAL in MNI using the TAL2MNI function available in GingerALE, we ended up with a set of coordinates that did not correspond any longer to the set of activations reported in the original study. Finally, we also considered as inclusion criteria: (5) only patients diagnosed with either UWS or MCS; (6) reporting data of single patients. The entire selection procedure is shown in Figure 1, and the full list of screened records is available in the supplementary materials Table S3 (INCLUSION-EXCLUSION.xlsx). The main characteristics of the 22 selected experiments are reported in Table 1.

Here it is worth noting that in the selected studies, the data from different patients were not analysed with classical second-level random effects group techniques, but rather, the results were reported according to the classic multiple-single-subjects approach. Therefore, we created the input database to conform to this approach; in particular, we entered activation peaks for every single patient included in the study of interest, which showed significant neural activity in that specific region (the entire database is reported in the supplementary materials: Table S4). Moreover, it is worth noting that in 11 studies, the authors did not explicitly report the stereotactic coordinates, but rather the anatomical label of the activated region. In these latter cases, we extracted from the Automatic Anatomical Labelling (AAL) [9] a representative stereotactic coordinate (see Table 2 for more details).

Using this approach, we selected a pool of 537 foci of activation distributed as follows: 441 foci were related to passive tasks, while 96 emerged from active tasks; 297 represented activity in the UWS patients, 240 represented the residual neural activity in MCS patients. The distribution of the activation peaks is reported in Table 3.

### 2.2. Meta-Analytic Procedures

The identification of the activations associated with active and passive tasks in DOCs were computed by means of the software GingerALE version 2.3.6 [34,35,36]. In particular, before starting with the meta-analytic procedure, we transformed all the stereotactic coordinates to the same stereotactic space (the MNI space) and in the neurological convention. The overall database was divided to map (a) the activations associated with active and passive tasks, (b) the activations specific for each group of patients, i.e., UWS and MCS. Once obtained the 4 sets of MNI coordinates, we applied the following procedure to the neurofunctional data:

1. anatomical masking—we applied a filtering of the coordinates using the “less conservative” mask available in the GingerALE software. After this procedure, 5/441 peaks belonging to the passive tasks and 2/96 belonging to the active tasks fell out of the mask. When running the analyses on the classes of patients, 1/240 activation peak was excluded from the MCS, 6/297 peaks were excluded from the UWS; therefore, the final overall number of foci was 523;
2. creation of the ALE maps—for this step we adopted a standard 6-mm full-width at half-maximum (FWHM) to model the Gaussian function around each single coordinate, according to the procedure reported in the methodological paper by Eickhoff [35];
3. thresholding procedure—the ALE maps were thresholded at *p* < 0.001 uncorrected in order to detect also the lowest level of activation across patients (for the sake of completeness, in the supplementary materials Table S2 we report also the results with the most conservative thresholding method available in the literature, i.e., *p* < 0.05 FWE).

As a final step, we obtained 4 ALE maps: one for the active tasks, one for the passive tasks, one for the UWS patients, one for the MCS. We then performed two different contrast analyses with the procedure implemented in GingerALE by contrasting active and passive tasks, as well as UWS and MCS. With this procedure, we were able to identify:

(a) the regions commonly activated by active and passive tasks (active–passive conjunction map) as well as the regions mostly activated by one condition (i.e., active tasks) as compared with the other one (i.e., passive tasks) and vice versa—these computations allowed us to test hypothesis 1;
(b) the regions commonly activated by UWS and MCS (UWS MCS conjunction map), as well as the regions mostly activated by one category of patients (i.e., MCS) as compared with the other one (i.e., UWS) and vice versa—these computations allowed us to test hypotheses 2 and 3.

Moreover, as reported in hypothesis 2, we are expecting to find a significant overlap between the dorsal-attentional network and the MCS map, while UWS patients, being unresponsive to external stimuli, by definition, should not activate this network. To explicitly test this assumption, we extracted the dorsal-attentional uniformity map from the neurosynth toolbox (www.neurosynth.org) by searching the term “dorsal-attentional”. The UWS and MCS ALE maps were binarised into VOIs using the software MRIcron [37]. We then applied a standard intersection analysis to identify the regions of anatomical overlap between each category of patients and the dorsal-attentional network. The intersection results were saved as VOIs and overlapped to the AAL template. By means of the “descriptive option” available in MRIcron we then performed a regional voxel count (as a consequence, all the results of the voxel-count analyses are re-scaled to 1 mm^3^). Finally, according to hypothesis 3, if UWS patients have a severer deficit of consciousness than MCS, then we should expect to find activations during passive tasks, on average, in both groups, while only MCS should manifest activations during active tasks. Accordingly, there might be a significant overlap between the UWS and MCS conjunction map, but this overlap should be located in areas associated with passive tasks only. To explicitly test this hypothesis, the UWS and MCS conjunction map was binarised into VOIs using the software MRIcron and overlapped with both the passive, and the active tasks ALE maps. Also, in this case we applied a classic voxel-count approach.

## 3. Results

The detailed anatomical description of the results is reported in Table 4 and Table 5, in what follows, we describe the main findings for every single effect of interest.

- Passive Task (Table 4): from the set of passive task activation peaks, we found a significant effect in the orbital cortices (BA 47) and in the inferior frontal gyri (pars triangularis; BAs 46, 45), at the border with the middle frontal gyrus (BA 9), in the lateral temporal cortices as well in subcortical regions. In particular, the two subcortical clusters were located (i) between the amygdala, the globus pallidus on the right hemisphere, and (ii) between the amygdala and the parahippocampal gyrus of the left hemisphere;
- Active Task (Table 4): from the pool of active task-related foci, significant clusters were found in the paracentral lobule (BAs 4 and 6), in the inferior parietal lobule (BA 40), in the fusiform gyrus (BA 20), in the lingual gyrus (BA 19), in the middle occipital gyrus (BA 19), in the superior occipital gyrus (BA 18), and in the cuneus (BA 18) of both hemispheres. Concerning the left hemisphere, we found a significant effect in the superior frontal gyrus (BA 8), in the inferior frontal gyrus pars triangularis (at the border with the middle frontal gyrus; BA 9), in the postcentral gyrus (BA 2), and in a left subcortical cluster located between the amygdala and the putamen nucleus. Moreover, we found a significant effect in the right middle frontal gyrus (BAs 8, 9, and 10);
- Passive and Active task - conjunction and contrast analysis: the active and passive map (Table 5) was computed to explicitly test hypothesis 1. As clearly reported in Table 5, the only brain region commonly activated by active and passive paradigm is the subcortical cluster located between the left amygdala and the putamen. Finally, the contrast analyses (*p* < 0.001 uncorrected) revealed a higher level of activation for the comparison “active > passive tasks” in the left paracentral lobule (x = −6, y = −24, z = 68; BA 6); the reversed contrast, namely “passive > active tasks” did not show any significant effect;
- Unresponsive Wakefulness Syndrome (UWS; Table 4): the patients in UWS showed significant activations in the inferior frontal gyrus, both in the pars orbitalis (BA 47) and in the pars triangularis (45, and 46) extending to the border with the middle frontal gyrus (BA 9), in the supplementary motor area (SMA; BA 6), in the lateral temporal cortices, and the amygdala (BA 34) of both hemispheres. Furthermore, a significant effect was found at the subcortical level between the amygdala, the globus pallidus on the right hemisphere and between the amygdala and the parahippocampal gyrus of the left hemisphere;
- Minimally Conscious State (MCS; Table 4): we found significant clusters of activations in the orbital part of the inferior frontal gyrus (BA 47), in the lateral temporal cortices, and in the fusiform gyrus (BA 20) of both hemispheres. Also, in this case, a significant effect was found at the subcortical level between the amygdala, the globus pallidus on the right hemisphere and between the amygdala and the parahippocampal gyrus of the left hemisphere. Furthermore, significant results emerged in the left inferior frontal gyrus at the border with the middle frontal gyrus (pars triangularis; BAs 9 and 45);
- UWS and MCS patients—conjunction and contrast analysis: the activations described across studies and shared by the two classes of patients were located in the orbital portions of the inferior frontal gyrus (BA 47), in the superior temporal gyrus (BA 41), and in the two subcortical clusters located around the amygdala of both hemispheres (Table 5). Furthermore, significant shared activations were found in the dorsal part of the left inferior frontal gyrus (at the border with the middle frontal gyrus; BAs 9 and 45), in the left middle temporal gyrus (BA 22), and in the right heschl gyrus (at the border with the insular cortex; BA 13). The between-groups contrast analyses did not show any significant result.

The results described at points “d”, “e” and “f” were computed to test hypothesis 2 and were further explored to describe the amount of overlap with the dorsal-attentional network. Contrary to our hypothesis, UWS patients activated part of the dorsal-attentional network, in particular at the level of the orbital portions of the inferior frontal gyrus, in the heschl gyrus, and in the superior temporal gyrus bilaterally, in the left triangular part of the inferior frontal gyrus, in the left inferior frontal operculum, in the left SMA, in the left precentral gyrus, in the left middle temporal gyrus, and in the right rolandic operculum (Table 6). Similarly, the MCS patients showed significant clusters of activations within the dorsal-attentional network in the heschl gyrus, in the superior temporal gyrus, in the fusiform gyrus bilaterally, in the left triangular part of the inferior frontal gyrus, in the left frontal operculum, in the left precentral gyrus, in the left middle temporal gyrus, in the right orbital portion of the inferior frontal gyrus, and in the right rolandic operculum (Table 6). The distribution of the activated voxels within the dorsal-attentional network across the two categories of patients was different (Chi^2^_(1)_ = 242.03; *p* < 0.001—Monte Carlo simulation with 100,000 iterations). 

**Figure 2:**
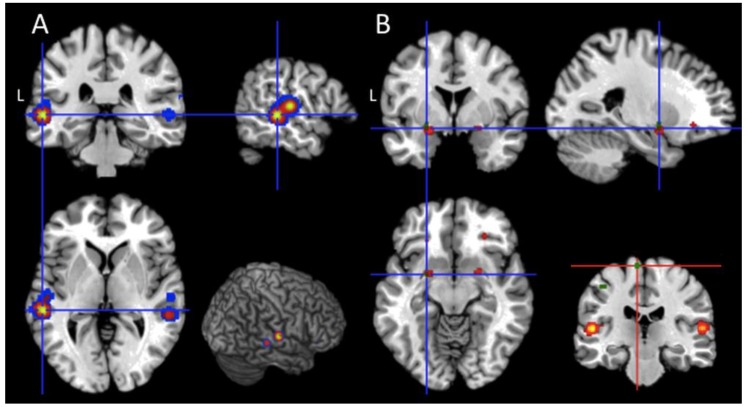
Intersection between patients and task category. The red regions represent the UWS and MCS map. (**A**) Overlap between UWS and MCS map (in red) and the passive maps (in blue; *p* < 0.001 uncorrected) at the level of the left superior and middle temporal gyri; the three-dimensional render shows the overlap at the level of the right lateral temporal cortices. (**B**) Anatomical overlap between the UWS and MCS map (in red) and the active map (in green; *p* < 0.001 uncorrected). The blue cross is centred over the left amygdala. Finally, the red cross depicted in the coronal section at the bottom of panel B is centred over the left paracentral lobule, which is specifically associated with “active tasks”.

Finally, to explicitly test hypothesis 3, we overlap the UWS and MCS maps, both with the passive and with the active maps. Interestingly, the majority of the brain regions reported in the UWS and MCS map belonged to the ALE map of the passive task (7492 out of the 7556 voxels belonged to the UWS and MCS map; Figure 2A), while only 48 out of the 7556 voxels included in the UWS and MCS map fell within the active task map; these voxels were located around the left amygdala (Figure 2B, blue cross).

## 4. Discussion

The clinical classification of DOC patients in UWS and MCS stems from the use of standardised scales, such as the CRS-R [4,5], which are based on the detection of behavioural signs such as purposeful motor responses. However, neuroimaging techniques combined with active paradigms could reveal signs of covert consciousness in unresponsive patients. Specifically, acquiring neuroimaging signals during active tasks may complement behavioural tools in assessing consciousness in DOC patients [1,7] in the clinical settings.

With this meta-analysis we (1) pooled the empirical efforts performed by different laboratories (2) to explicitly test three working hypotheses concerning the neurofunctional differences and commonalities between UWS and MCS patients engaged in active and passive tasks. In what follows we will discuss the results in the light of these working hypotheses.

### 4.1. Active and Passive Task-Related Activations in DOC Patients

Seventeen out of the 22 studies included in this meta-analysis used only passive tasks; most of these studies adopted either a passive linguistic or an emotional stimulation. Only 3 studies adopted a purely active paradigm, while 2 reported activations both for active, both for passive tasks. We are aware that the imbalance in the raw data might have affected the results of our meta-analysis. Nevertheless, despite this clear imbalance, we found a higher number of activation clusters for active tasks than for passive tasks, although these latter covered a larger brain volume as a whole (brain volume in passive tasks: 16,520 mm^3^; brain volume in active tasks: 3336 mm^3^, see Table 4), and with an anatomical distribution that appears to be consistent with our first working hypothesis: the active tasks induce a more widespread activation within the associative regions such as the dorsal PFC, the left inferior parietal and paracentral lobules, along with the medial structures of both the occipital and temporal lobes. Interestingly, when querying the Neurosynth toolbox with the stereotactic coordinates reported in Table 4 for active tasks, the local maxima of the cluster centred over the paracentral lobule was associated with the term motor imagery (posterior probability = 0.86) suggesting an engagement of cerebral regions involved in the active processing of mental representations. 

On the contrary, passive tasks were associated with a significant activation of the inferior frontal, of the superior and middle temporal cortices, i.e., with brain regions that are typically related with linguistic processing (14 out the 19 studies that used at least one passive task condition). Interestingly, the only cluster that, according to a formal contrast analysis, was commonly activated by active and passive tasks is located in a left subcortical region at the border between the amygdala and the putamen nucleus: a region that, according to the Neurosynth database, is associated with “pictures” processing (posterior probability = 0.75) and with “olfactory” processing (posterior probability = 0.85). If one turns back to the input database, this brain regions was reported in the study by Nigri et al. [31], in which an olfactory stimulation was used, in the study by Liang et al. [14] (x = −24.6, y = −0.9, z = −7.26), in which a mental imagery task was adopted. To conclude, the results of our meta-analysis suggest that active and passive paradigms map, overall, a pool of dissociable brain regions, at least from the neurofunctional point of view with a unique “conjunction point” at the subcortical level.

### 4.2. Are There Neurofunctional Markers of Consciousness Recovery in the dorsal-attentional Network?

As clearly pointed out by Posner, “*within neurology, consciousness often refers to a brain state in which the person is capable of responding to external events and relate them to the self. This state is closely associated with the concept of arousal and to the diurnal cycle of sleep and wake*” [38] (p. 1); moreover, often the term consciousness is associated with the construct “attention”, under the somehow simplistic assumption that, if one can guide his/her attention toward an external stimulus, then he/she must be conscious (but see also [39] for examples of attention without consciousness). This skill is associated with the functions of the dorsal-attentional network, the network responsible for externally directed cognitive processes [40], which mediates cognitive responses elicited by external stimuli. According to the clinical definition of “unresponsive wakefulness syndrome” *(“patients who show several clinical signs (hence the use of syndrome) of unresponsiveness (meaning they failed to show non-reflex behaviour or command following) in the presence of wakefulness (meaning they open their eyes spontaneously or upon stimulation)”*) by Laureys et al. [2] (pp. 2–3), in these patients the manifestation of cognitive/behavioural responses elicited by external stimuli should be significantly affected and hence, one could assume that the activity of the underlying neural network, i.e., the dorsal-attentional network, should be significantly reduced. On the contrary, in patients with minimal signs of consciousness recovery (i.e., in MCS patients), the neurofunctional activity of this network should be at least partially preserved. We wanted to explore whether UWS patients manifested a significant reduction of activation within the dorsal-attentional network when compared with MCS patients, and we found that results did not seem to support this hypothesis. At the global level (see Table 7 for more details), in fact, the empirical findings are in opposition with our hypothesis as the proportion of voxels stored in the UWS maps that fall within the dorsal-attentional (5.5%) is basically the same of the proportion of MCS-related activations within the same brain regions (5.9%; Chi^2^_(1)_ = 1.583, *p* = 0.208).

Notwithstanding these similarities at the global level, the distribution of the data at the regional levels showed some interesting findings (see Table 6). While some regions of the DAN showed exactly the same activation extent between the two groups of patients (namely the left inferior orbital gyrus, the left rolandic operculum, the left precentral gyrus, the left inferior frontal gyrus pars triangularis), some other regions, like, for example, the left and right fusiform gyri, were exclusively activated by either MCSs or by UWS.

Taken together, the results of our analysis do not support the idea that responding to external stimuli indicates the presence of “consciousness”; rather, they suggest that the partial sparing of a specific network, such as the dorsal-attentional network, does not represent a sufficient condition to clinically preserve island of consciousness; neither is a necessary condition for classifying a patient as “conscious”. We agree that the definition of the conscious state is difficult, and it has also been conceptualised as the consequence of the interplay between different processes/dimensions. For instance, Laureys [3] suggested that the level of consciousness (ranging from “*coma*” to “*conscious wakefulness*”) depends on the interaction between the level of wakefulness and the level of awareness. Similarly, Dehaene et al. [41] hypothesised that the interactions between bottom-up (i.e., stimulus strength) and top-down (i.e., attentional modulation) processes could determine the level of consciousness. More generally, Tononi and Koch suggested that consciousness requires that functionally specialised modules of the thalamocortical system interact rapidly and effectively [42].

These different processes/dimensions should involve multiple neural networks. Future studies combining behavioural and neuroimaging techniques should test these hypotheses more systematically. However, our results support the idea proposed by Gaillard and colleagues: “*rather than hoping for a putative unique marker (the neural correlate of consciousness), a more mature view of conscious processing should consider that it relates to a distributed pattern of brain activation that occurs at a specific level within a complex anatomical and functional architecture*” [43] (p. 0489).

### 4.3. The Minimum State of the Brain: Shared Activations between UWS and MCS

Patients diagnosed with UWS are characterised by a complete unawareness of the self and of the environment, while patients in MCS manifest a variable degree of self and environmental awareness [2,44,45]. Thus, the common clinical feature of these 2 classes of patients is a certain level of arousal maintenance, with responses to passive stimulations not necessarily associated with volition. Accordingly, we divided the available fMRI tasks into two well-segregated categories on the basis of the required patients’ commitment to the task. In particular, we defined as active those tasks in which patients were explicitly required to perform a command following in the absence of passive external stimuli (apart from the command itself), while passive tasks were all those tasks in which patients received either auditory or visual stimuli without specific active-task instructions. This dichotomic classification allowed us not only to test their across-study neurofunctional differences, but also to look at what we could define as “the minimum state of the brain”, i.e., the minimum set of brain regions that is commonly activated by UWS and MCS, and that should be found in regions associated with passive task and not with active task. We found that the set of perisylvian brain regions commonly activated by UWS and MCS belongs to the passive task-maps, while there are only minimal overlaps within the active network at the subcortical level. This set of brain regions may represent the neurofunctional correlates of the minimum state of the brain in DOC patients that emerged from coma. Moreover, the fact that the proportion of activation peaks reported for MCS patients in active task (24.16%, see Table 3 for more details) doubles the proportion of UWSs’ activation peaks in the same category of tasks (12.79%, see Table 3 for more details) suggests that the UWS activations are mainly associated with passive response in perysilvian and sub-cortical regions, while MCS patients are able to recruit, at least in part, regions associated with mental imagery (neurosynth posterior probability = 0.7) and spatial navigation (neurosynth posterior probability = 0.8) such as the fusiform/parahippocampal regions reported in Table 4. 

The continuum between different conscious states has been widely discussed [3,41], and the cerebral activations of UWS have been explained as the result of either bottom-up processes [41] or interpreted as the neurophysiological correlate of wakefulness without awareness [3]. Conversely, MCS-specific activations in active tasks would reflect the involvement of either top-down processes [41], or the correlate of a partial recovery of awareness [3]. This could be particularly true if the shared activations in passive task by the two classes of patients would be limited to “low-level” cortical and subcortical regions. However, our patients did not share activations only in “low-level” cortical and subcortical regions, with the only exception being the primary cortical areas such as the heschl gyri. Nevertheless, we cannot exclude that the shared activation of higher-order cortices activated during passive task may derive from some level of inaccuracy either in the clinical classification of patients. To make a suggestive example, a patient in the study by Fernandez-Espejo et al. [23] was retrospectively classified as being UWS on the basis of the clinical rating. Interestingly, 8 out of 13 activation peaks retrieved from this study were derived from this patient. Furthermore, the activation peaks extracted from the study by Liang et al. [14] were derived from two patients who showed signs of consciousness, as also stated by the authors themselves (see [14] clinical description in the supplementary materials). In the same vein, the UWS and MCS patients in the study by Nigri et al. [30] manifested similar values at 5 out of the 6 subscales of the CRS-R; for example, in the auditory subscale, the mean rank in the group of UWS was 5.88, while MCS obtained 6.07 (Mann-Whitney U tests = 13.5, *p* = 0.9). The only significant between-groups difference was at the visual subscale in which the UWS patients obtained a mean rank of 2.88, while the MCS patients obtained 7.79 (Mann-Whitney U test = 1.5, *p* = 0.01). The problem of patients’ misclassification is also more intriguing if one looks at the studies that contributed to the clusters located in SMA for UWSs (namely clusters 8 and 9 in Table 4). In particular, the cluster located in the in the left SMA (cluster 8, Table 4) included 1 activation peak from the study by Bekinschtein et al. [18] and 4 coordinates from the study by Monti et al. [1]. Interestingly, in the study by Monti et al. [1], there was only a clinical classification of the patients, with a complete lack of standardised measures, such as the CRS-R, which would allow us to better appreciate their level of impairment. Meanwhile, cluster 9 (centred in the right SMA) included 1 activation peak from the study by Bekinschtein et al. [18] and 3 activation peaks from Qin et al. [21]. Interestingly, in this latter study, 2 out of the 7 UWS patients emerged from the vegetative state within 3 months after the fMRI session. The issue related to the fluctuation of the responsiveness in DOC patients was recently explored by Wannez et al. [46]; in this recent paper, the authors suggest a minimum of 5 consecutive assessments with the CRS-R to obtain a reliable classification of the patients.

To conclude, these incongruencies suggest that DOC patients can be better described along a clinical continuum rather than according to independent clinical categories. This continuum seems to better correspond to a hierarchical model of consciousness with different physiological/cognitive levels referring to specific neurofunctional networks.

## 5. Conclusion: Recommendations for Neuroimaging Studies of DOC Patients and Limitation of the Study

When approaching the effort of quantitatively reviewing a piece of scientific literature, several issues may arise. We want to conclude by reporting part of these issues and of the difficulties that we encountered in performing this meta-analysis with the aim of giving to the readers some suggestions that should be followed when reporting the results of neuroimaging data on DOC patients.

The first issue we encountered was related to the distinction between neurological and radiological conventions. Indeed, we found 12 out of 38 studies in which it was not explicitly stated whether the results reported either in the tables or in the figures were in radiological or in neurological convention. This represents an issue for coordinate-based meta-analyses, especially when the coordinates refer to another reference system (i.e., TAL instead of MNI), since once flipped along the y-axis, the correspondence between the MNI coordinates in the neurological convention and the original results (in TAL radiological convention) was not maintained.

Furthermore, 11 out of the 22 selected studies reported only the anatomical labels of the activated brain regions (see Table 2) as the results are computed in a single-subject anatomical space, apart from [30,33]. In these cases, it would have been useful to report, at the very least, information about the volumetric extension of each regional activation, for example, in terms of activated voxels, as this additional information could have helped us, and future studies, in estimating the anatomical distribution of the task-related fMRI results in each single patient. As a final suggestion, it would be useful to create a shared open-access fMRI database to store, at least, the basic clinical standardised scales (such as the CRS), a volumetric T1 image, together with a resting-state fMRI, to promote data sharing and pooled data analyses that, in the long run, could contribute to a better neurofunctional characterisation of patients with disorders of consciousness after severe brain damage.

As a final remark, we would like to make some limitations of the study explicit:

1. The GingerALE approach only permits the comparison of two categories and looking at their commonalities. This means that every other higher-order comparison, as well as comparisons with other maps (such as those extracted from Neurosynth) cannot be performed using an inferential approach. This is the reason we adopted a voxel-count approach for our intersection analyses. However, the readers should be aware that these analyses gave just information about the spatial extent (as expressed in terms of voxels) of anatomical overlap.
2. Some of the results reported here may be triggered by the specific set of experimental tasks implemented in the fMRI studies. Future meta-analyses should better evaluate task-specific effect in this literature.

## Figures and Tables

**Table 1:** Studies included in the meta-analysis. The table shows the main characteristics of the studies included in the meta-analysis.

Study ID	Authors	Year	Technique	Sample Size	Task Category	Task Type
1	Wang et al. ^∞^ [13]	2005	fMRI	66 (*n* = 39 UWS; *n* = 25 MCS; *n* = 2 EMCS)	passive	Spoken own name by a familiar voice
2	Liang et al. [14]	2014	fMRI	5 (*n* = 2 UWS; *n* = 3 MCS)	passive and active	Spoken sentences and motor/mental imagery
3	Monti et al. [15]	2013	fMRI	1 (MCS)	passive	Visual stimulation
4	Hampshire et al. ^χ^ [16]	2013	fMRI	1(UWS)	active	Visual imagery
5	Crone et al. [17]	2011	fMRI	25 (*n* = 17 UWS; *n* = 8 MCS)	passive	Sentences listening
6	Bekinschtein et al. ^α^ [18]	2011	fMRI	5 (all UWS)	passive	Hand movement verbal command
7	Moreno et al. ^Δ^ [19]	2010	fMRI	10 (*n* = 3 UWS; *n* = 5 MCS; *n* = 1 EMC; *n* = 1 LIS)	active	Visual naming
8	Fernández-Espejo et al. ^£^ [20]	2010	fMRI	1 (UWS)	passive	Sentences listening
9	Qin et al. [21]	2010	fMRI	11 (*n* = 7 UWS; *n* = 4 MCS)	passive	Spoken own names by familiar voice
10	Monti et al. ^•^ [1]	2010	fMRI	54 (*n* = 23 UWS; *n* = 31 MCS)	active	Motor imagery
11	Heelmann et al. [22]	2010	fMRI	6 (all UWS)	passive	Visual and sensory stimulations
12	Fernández-Espejo et al. [23]	2008	fMRI	7 (*n* = 3 UWS; *n* = 4 MCS)	passive	Narratives listening
13	Di et al. * [24]	2007	fMRI	11 (*n* = 7 UWS; *n* = 4 MCS)	passive	Spoken own name by a familiar voice
14	Staffen et al. [25]	2006	fMRI	1 (UWS)	passive	Spoken own name by a familiar voice
15	Bekinschtein et al. ^×^ [26]	2005	fMRI	1 (UWS)	passive	Sentences listening
16	Owen et al. ^µ^ [27]	2005	fMRI	1 (UWS)	passive	Sentences listening
17	Owen et al. [28]	2002	fMRI	3 (all UWS)	passive	Visual stimulation, familiar face perception, and speech perception
18	Sharon et al. [29]	2013	fMRI	4 (all UWS)	passive and active	Face perception and visual imagery
19	Nigri et al. ^¥^ [30]	2017	fMRI	14 (*n* = 4 UWS; *n* = 10 MCS)	passive	Words listening
20	Nigri et al. [31]	2016	fMRI	33 (*n* = 26 UWS; *n* = 7 MCS)	passive	Olfactory stimulation
21	Tomaiuolo et al. [32]	2016	fMRI	1 (tested twice both in UWS and MCS)	passive	Sentences listening
22	Kotchoubey et al. ^+^ [33]	2014	fMRI	55 (*n* = 29 UWS; *n* = 26 MCS)	passive	Sentences listening

Δ Moreno et al., 2010 [19]; + Kotchoubey et al., 2014 [33]; ¥ Nigri et al., 2017 [30]; • Monti et al., 2010 [1]; * Di et al., 2007 [24]; ∞ Wang et al., 2005 [13]; χ Hampshire et al., 2013 [16]; α Bekinschtein et al., 2011 [18]; £ Fernández-Espejo et al., 2010 [20]; × Bekinschtein et al., 2005 [26]; µ Owen et al., 2005 [27].

**Table 2:** Stereotactic coordinates. Activation peaks attributed to the neurofunctional results of the fMRI studies that did not explicitly report any stereotactic coordinate. The coordinates reported in the table refer to the template by Tzourio-Mazoyer et al. [9]. Different studies are marked with different symbols (according to the symbols adopted in Table 1).

Brain Regions	Study	Left Hemisphere			Study	Right Hemisphere		
		x	y	z		x	y	z
Precentral gyrus	^χ; α^	−39	−6	51				
Superior frontal gyrus	^Δ^	−18	35	42				
Middle frontal gyrus	^Δ^	−33	33	35	^Δ^	38	33	34
					^Δ^	22	31	44
Inferior frontal gyrus	^+^	−46	30	14	^+^	50	30	14
	^+^	−36	31	−12	^¥; +^	41	32	−12
	^¥^	−36	31	12				
Supplementary Motor Area	^•; α^	−5	5	61	^α^	9	0	62
Inferior parietal	^Δ^	−43	−46	47	^Δ^	46	−46	50
Paracentral lobule	^Δ^	−8	−25	70	^Δ^	7	−32	68
Postcentral gyrus	^Δ^	−42	−23	49				
Heschl gyrus	*^; ∞; ×^	−42	−19	10	*^; ∞^	46	−17	10
Superior temporal gyrus	*	−56	−38	18	*	56	−38	18
	*	−41	−39	18	*	53	−21	3
	*	−53	−21	3	^+; ¥; ∞; α; µ^	58	−22	7
	^+; ¥; ∞; α; £; ×; µ^	−53	−21	7				
Middle temporal gyrus	*^; +; ¥; α; £; µ^	−56	−34	−2	*^; +; ¥; α; µ^	57	−37	−1
Insula					^χ^	39	6	2
Parahippocampal	^•^	−21	−16	−21	^•; χ^	25	−15	−20
Superior occipital gyrus	^Δ; χ^	−17	−84	28	^Δ^	24	−81	31
Middle occipital gyrus	^Δ^	−32	−81	16	^Δ^	37	−80	19
Fusiform	^Δ^	−31	−40	−20	^Δ^	34	−39	−20
Lingual	^Δ^	−15	−68	−5	^Δ^	16	−67	−4
Cuneus	^Δ^	−6	−80	27	^Δ^	14	−79	28

Δ Moreno et al., 2010 [19]; + Kotchoubey et al., 2014 [33]; ¥ Nigri et al., 2017 [30]; • Monti et al., 2010 [1]; * Di et al., 2007 [24]; ∞ Wang et al., 2005 [13]; χ Hampshire et al., 2013 [16]; α Bekinschtein et al., 2011 [18]; £ Fernández-Espejo et al., 2010 [20]; × Bekinschtein et al., 2005 [26]; µ Owen et al., 2005 [27].

**Table 3:** Distribution of the activation peaks. Here we report the patient-by-task category contingency table and the resulting test statistic (chi-squared test).

		Patients Category		Total
		*UWS*	*MCS*	
**Tasks Category**	active	38	58	96
	passive	259	182	441
	Total	297	240	537

Chi^2^_(1)_ = 11.69, *p* = 0.001; UWS = unresponsive wakefulness syndrome; MCS = Minimally Conscious State.

**Table 4:** Clusters of activation. The clusters of activation are reported for each task (both passive and active) and for each patients’ category (both unresponsive wakefulness syndrome and minimally conscious state). The brain regions are labelled according to the Automatic Anatomical Labelling (AAL [23]). Double labels are reported for those regions presenting a different anatomical location according to the mapping procedure implemented in GingerALE [34,35,36].

Cluster’ Number	Area	x	y	z	Brodmann Area	Volume (mm^3^)
**Passive Tasks**						
1	Left Inferior Frontal Gyrus—triangular part/Middle Frontal Gyrus	**−50**	**16**	**30**	9	56
2	Right Inferior Frontal Gyrus—triangular part/Middle Frontal Gyrus	**49.8**	**30.2**	**14.4**	46	72
3	Left Inferior orbito-frontal	**−24**	**30**	**−10**	47	56
4	Left Inferior orbito-frontal	**−36**	**31**	**−12**	47	16
5	Left Inferior Frontal Gyrus—triangular part	**−44.3**	**25.8**	**12.9**	45	384
6	Right Inferior orbito-frontal	**40.9**	**32.1**	**−12**	47	496
7	Right Inferior orbito-frontal	**28**	**34**	**−12**	47	56
8	Left Superior Temporal Gyrus	**−54.8**	**−25.7**	**3.8**	41	8912
	Left Middle Temporal Gyrus	*−56*	*−34*	*−2*		
	Left Heschl Gyrus/Insula	*−42*	*−20*	*10*	13	
9	Right Superior Temporal Gyrus	**56.7**	**−20.5**	**8.2**	41	4192
	Right Heschl Gyrus/Insula	*46*	*−18*	*10*	13	
	Right Thalamus/Superior Temporal Gyrus	*8*	*−8*	*4*	22	
	Right Superior Temporal Gyrus	*68*	*−28*	*16*	42	
10	Right Middle Temporal Gyrus	**68.5**	**−18**	**−8.8**	21	120
11	Right Middle Temporal Gyrus	**57.2**	**−36.8**	**−1**	22	1712
12	Left Amygdala/Parahippocampal Gyrus	**−22.3**	**−0.3**	**−14.7**	34	296
13	Right Amygdala/Globus Pallidus	**22.5**	**1.8**	**−12.6**		152
**Active Tasks**						
1	Right Middle Frontal Gyrus/Superior Frontal Gyrus	**33**	**53**	**11**	10	64
2	Right Middle Frontal Gyrus/Superior Frontal Gyrus	**20.6**	**32**	**43**	8	208
3	Left Inferior Frontal Gyrus—triangular part/Middle Frontal Gyrus	**−33**	**33**	**35**	9	64
4	Right Middle Frontal Gyrus	**38**	**33**	**34**	9	80
5	Left Superior Frontal Gyrus	**−18**	**35**	**42**	8	80
6	Left Paracentral Lobule	**−7.2**	**−24.6**	**70**	6	136
7	Right Paracentral Lobule	**6.9**	**−31.5**	**68.1**	4	104
8	Left Postcentral Gyrus	**−42**	**−23**	**49**	2	96
9	Left Inferior Parietal Lobule	**−43.8**	**−45.5**	**45.3**	40	200
10	Right Inferior Parietal Lobule	**46**	**−46**	**50**	40	56
11	Left Fusiform Gyrus	**−31**	**−40**	**−20**	20	80
12	Right Fusiform Gyrus	**31.7**	**−38.9**	**−21.1**	20	1224
13	Left Lingual Gyrus	**−15**	**−68**	**−5**	19	96
14	Left Middle Occipital Gyrus	**−32**	**−81**	**16**	19	80
15	Right Middle Occipital Gyrus	**37**	**−80**	**19**	19	96
16	Left Superior Occipital Gyrus/Cuneus	**−17**	**−84**	**28**	18	224
17	Left Cuneus	**−6.5**	**−80**	**27**	18	112
18	Right Superior Occipital Gyrus/Cuneus	**24**	**−81**	**31**	18	96
19	Right Cuneus	**14**	**−79**	**28**	18	80
20	Left Amygdala/Putamen	**−24.4**	**−1**	**−10.8**		80
21	Right Lingual Gyrus	**16**	**−67**	**−4**		80
**Unresponsive Wakefulness Syndrome**						
1	Left Inferior Frontal Gyrus—triangular part/Middle Frontal Gyrus	**−50**	**16**	**30**	9	56
2	Right Inferior Frontal Gyrus—triangular part/Middle Frontal Gyrus	**49.8**	**30.2**	**14.4**	46	72
3	Left Inferior orbito-frontal Gyrus	**−24**	**30**	**−10**	47	56
4	Left Inferior orbito-frontal Gyrus	**−36**	**31**	**−12**	47	16
5	Left Inferior Frontal Gyrus—triangular part	**−44.3**	**25.8**	**12.9**	45	384
6	Right Inferior orbito-frontal Gyrus	**40.9**	**32**	**−12**	47	488
7	Right Inferior orbito-frontal Gyrus	**28**	**34**	**−12**	47	56
8	Left Supplementary Motor Area	**−4.3**	**4.5**	**60.5**	6	160
9	Right Supplementary Motor Area	**7.7**	**0.6**	**63.3**	6	184
10	Left Superior Temporal Gyrus	**−54.4**	**−25.3**	**4.3**	41	7560
	Left Middle Temporal Gyrus	*−56*	*−34*	*−2*		
	Left Heschl Gyrus/Insula	*−42*	*−20*	*10*	13	
11	Right Superior Temporal Gyrus	**55.6**	**−20.7**	**8**	41	3096
	Right Heschl Gyrus/Insula	*46*	*−18*	*10*	13	
12	Right Middle Temporal Gyrus	**68.4**	**−17.8**	**−8.7**	21	112
13	Right Middle Temporal Gyrus	**57.5**	**−36.9**	**−1**	22	1552
14	Left Amygdala/Parahippocampal Gyrus	**−22.7**	**−0.3**	**−13.8**	34	536
15	Right Amygdala/Globus Pallidus	**22.6**	**1.9**	**−12.8**		144
**Minimally Conscious State**						
1	Left Inferior Frontal Gyrus—triangular part/Middle Frontal Gyrus	**−50**	**16**	**30**	9	56
2	Left Inferior orbito-frontal Gyrus	**−24**	**30**	**−10**	47	56
3	Left Inferior Frontal Gyrus—triangular part	**−43.6**	**21.9**	**12**	45	128
4	Right Inferior orbito-frontal Gyrus	**28.2**	**34.6**	**−12.2**	47	96
5	Left Fusiform Gyrus	**−30.8**	**−40.2**	**−19.8**	20	248
6	Right Fusiform Gyrus	**31.8**	**−39.1**	**−21**	20	624
7	Left Superior Temporal Gyrus	**−53.8**	**−26.3**	**3.1**	41	4576
	Left Middle Temporal Gyrus	*−56*	*−34*	*−2*		
	Left Heschl Gyrus/Insula	*−42*	*−20*	*10*	13	
8	Right Superior Temporal Gyrus	**58.3**	**−22**	**7.4**	41	1248
9	Right Middle Temporal Gyrus	**56.7**	**−37**	**−1**	22	488
10	Right Heschl Gyrus/Insula	**46.2**	**−17.2**	**10.1**	13	592
11	Left Amygdala/Parahippocampal Gyrus	**−22.4**	**−0.4**	**−14.8**	34	360
12	Right Amygdala/Globus Pallidus	**22.4**	**2**	**−12.6**		192

**Table 5:** Conjunction results. Brain areas of shared activation obtained by means of the contrast analysis procedure implemented in GingerALE [34,35,36] at *p* < 0.001 uncorrected (the FWE-corrected results are reported in Table S1 of the supplementary materials).

Cluster Number	Area	x	y	z	Brodmann Area	Volume (mm^3^)
**Active and Passive**						
1	Left Amygdala/Putamen	**−24**	**−1**	**−13**		32
**UWS and MCS**						
1	Left Inferior orbito-frontal	**−24**	**30**	**−10**	47	56
2	Right Inferior orbito-frontal	**28**	**34**	**−12**	47	56
3	Left Inferior Frontal Gyrus—triangular part/Middle Frontal Gyrus	**−50**	**16**	**30**	9	56
4	Left Inferior Frontal Gyrus—triangular part	**−43.7**	**22.1**	**12.1**	45	112
5	Left Superior Temporal Gyrus	**−53.7**	**−26.3**	**3.3**	41	4432
	Left Middle Temporal Gyrus	*−56*	*−34*	*−2*		
	Left Heschl Gyrus/Insula	*−42*	*−20*	*10*	13	
6	Right Superior Temporal Gyrus	**58.3**	**−22**	**7.4**	41	1248
7	Right Heschl Gyrus/Insula	**46.2**	**−17.2**	**10.1**	13	592
8	Left Middle Temporal Gyrus	**−56.7**	**−37**	**−1**	22	488
9	Left Amygdala/Parahippocampal Gyrus	**−22.4**	**−0.4**	**−14.8**	34	360
10	Right Amygdala/Globus Pallidus	**22.6**	**1.9**	**−12.8**		144

**Table 6:** Intersection results within the dorsal-attentional network. Brain areas of intersection (first column) and number of voxels in both unresponsive wakefulness syndrome patients (second column) and minimally conscious state patients (third column) obtained by means the voxel-count approach available in MRIcron [37].

Brain Region	Voxel Count	
	UWS	MCS
Left frontal inferior triangular part	41	41
Left Inferior orbito-frontal	4	0
Right Inferior orbito-frontal	10	10
Left frontal inferior operculum	2	2
Right Rolandic operculum	25	8
Left supplementary motor area	124	0
Left precentral gyrus	1	1
Left Heschl gyrus	46	30
Right Heschl gyrus	19	13
Left superior temporal	67	35
Right superior temporal	362	225
Left middle temporal	97	53
Left fusiform gyrus	0	90
Right fusiform gyrus	0	6

**Table 7:** Distribution of activation peaks within the dorsal-attentional network. The number of peaks is indicated for each patients’ category either within or outside the dorsal-attentional network. The total number of voxels represents the volume extent of the UWS and MCS conjunction map. These voxels are classified according to the intersection with the dorsal attentional network uniformity map. Under the table: the chi-squared test on the distribution of the activation peaks. UWS = unresponsive wakefulness syndrome; MCS = minimally conscious state; DAN = dorsal-attentional network.

		Patients’ Category		Total
		*UWS*	*MCS*	
**DAN**	Yes	798	514	1312
	No	13,662	8178	21,840
	Total	14,460	8692	23,152

Chi^2^_(1)_ = 1.583, *p* = 0.208.

## Supplementary Materials

The following are available online at https://www.mdpi.com/2077-0383/8/4/516/s1, Table S1: Clusters of activation FWE corrected. Table S2: Conjunction results FDR corrected. Table S3: INCLUSION-EXCLUSION.xlsx. Table S4: ACTIVATIONS.xlsx.
