# Supplementary material for: Neuroimaging Studies on Disorders of Consciousness: A Meta-Analytic Evaluation
Source: J Clin Med. 2019 Apr 16;8(4):516. doi: 10.3390/jcm8040516 (PMC6517954; doi:10.3390/jcm8040516)
Supplement: Supplementary file 1 [file jcm-08-00516-s001.zip › Tables S1_S2.docx]

**Table S1. Clusters of activation FWE corrected.** The clusters of activation are reported for each task (both passive and active) and for each patients’ category (both unresponsive wakefulness syndrome and minimally conscious state).

| **Cluster #** | **Area** | **x** | **y** | **z** | **Brodmann Area** | **Volume (mm^3^)** |
| --- | --- | --- | --- | --- | --- | --- |
| ***Passive Tasks*** | | | | | | |
| 1 | Left Superior Temporal Gyrus | **-53.9** | **-21.2** | **7.2** | 41 | 640 |
| 2 | Right Superior Temporal Gyrus | **58.1** | **-21.9** | **7.1** | 41 | 344 |
| 3 | Left Middle Temporal Gyrus | **-56.8** | **-33** | **-1.5** |  | 320 |
| 4 | Right Middle Temporal Gyrus | **57** | **-37** | **-1** | 22 | 64 |
| ***Active Tasks*** | | | | | | |
| 1 | Right Fusiform Gyrus | **32** | **-39** | **-21** | 20 | 96 |
| ***Unresponsive Wakefulness Syndrome*** | | | | | | |
| 1 | Left Superior Temporal Gyrus | **-54** | **-20.9** | **7.3** | 41 | 744 |
| 2 | Right Superior Temporal Gyrus | **58** | **-21.7** | **7.1** | 41 | 416 |
| 3 | Left Middle Temporal Gyrus | **-56.9** | **-33.2** | **-1.6** |  | 360 |
| 4 | Right Middle Temporal Gyrus | **57** | **-37** | **-1** | 22 | 64 |
| ***Minimally Conscious State*** | | | | | | |
| 1 | Left Superior Temporal Gyrus | **-53.1** | **-21.3** | **6.9** | 41 | 288 |
| 2 | Right Superior Temporal Gyrus | **58** | **-22** | **7** | 41 | 80 |
| 3 | Left Middle Temporal Gyrus | **-56.7** | **-32.8** | **-1.4** |  | 224 |

**Tables2. Conjunction results FDR corrected.** Brain areas of shared activation obtained by means of the contrast analysis procedure implemented in GingerALE

| **Cluster #** | **Area** | **x** | **y** | **z** | **Brodmann Area** | **Volume (mm^3^)** |
| --- | --- | --- | --- | --- | --- | --- |
| ***UWS & MCS*** | | | | | | |
| 1 | Left Superior Temporal Gyrus | **-53.1** | **-21.3** | **6.9** | 41 | 288 |
| 2 | Right Superior Temporal Gyrus | **58** | **-22** | **7** | 41 | 80 |
| 3 | Left Middle Temporal Gyrus | **-56.7** | **-32.8** | **-1.4** |  | 224 |
| ***Passive > Active*** | | | | | | |
| 1 | Left Superior Temporal Gyrus | **-53.9** | **-21.3** | **7.2** | 41 | 640 |
|  | Left Superior Temporal Gyrus | *-52* | *-18* | *9.3* | 13 |  |
| 2 | Right Superior Temporal Gyrus | **58.1** | **-22** | **7.1** | 41 | 344 |
|  | Right Superior Temporal Gyrus | **58** | **-18** | **6** | 41 |  |
| 3 | Left Middle Temporal Gyrus | **-56.8** | **-33** | **-1.5** |  | 320 |
|  | Left Middle Temporal Gyrus | **-58** | **-32** | **-0.6** | 21 |  |
| 4 | Right Middle Temporal Gyrus | **57** | **-37** | **-1** | 22 | 64 |
